# A Case of Puerperal Convulsions Effectually Controlled by the Inhalation of Chloroform

**Published:** 1867

**Authors:** John W. Hensley

**Affiliations:** Yates City, Knox Co., Ill.


### *A Case of Puerperal Convulsions Effectually Coutrolled by the Inhalation of Chloroform.* By John W. Hensley, M.D., of Yates City, Knox Co., Ill.

Was called to see Mrs. A., primapara, Thursday evening, March 7th, 1867. Upon arriving at her bedside, found her struggling in a violent convulsion—eyes rolled upwards; head twisted to one side; a bloody frothing at the mouth; and the extremities affected with clonic spasm. The eclampsia lasted some fifteen minutes, leaving the patient wholly unconscious; breathing stertorous and accompanied with occasional deep moaning. Was informed that labor commenced at noon, the dap previous; the convulsions first began at 9 A.M. on the 7th, and had already lasted twelve hours, occurring every few minutes. Her physicians, after attempting to take blood from the arm, I was told, had abandoned the case, as hopeless, half-an-hour before I arrived.

On examination, found the liquor amnii evacuated, os uteri perfectly dilated, and the child's breech presenting; the pelvis was rather narrow, and the patient seemed almost moribund. Yet, I conld see no good reason for abandoning the case without further effort. Recalled Dr. Baily to the bedside, suggested the administration of chloroform and the use of instruments to effect the delivery. Suffice it to say, the convulsions were effectually controlled by the chloroform, and the child, by means of the forceps and the blunt hook, extracted in the course of two hours. The uterus contracted at regular intervals, and the placenta was removed without trouble. The child, still-born, had been dead, apparently, for some hours. Patient still unconscious; breathing labored and loud. Four hours after discontinuing the chloroform the convulsions returned; had three, though lighter than they had been. Again administered the anæsthetic, with perfect and permanent success. The patient remained unconscious for about two days, with stertorous breathing; tongue very much swollen; had some trouble to control the uterine hemorrhage; retention of urine for a week, except when drawn off by the catheter. The patient is now about well.

*Remarks.*—I report this case, to show the value of chloroform in controlling puerperal eclampsia, for I am satisfied that Mrs. A. would have died in spite of every effort to save her life, had it not been for the timely use of this remedy.

